# Trichlorido{2-[2-(η^5^-cyclo­penta­dien­yl)-2-methyl­prop­yl]-1-trimethyl­silyl-1*H*-imidazole-κ*N* ^3^}titanium(IV) tetra­hydro­furan hemisolvate

**DOI:** 10.1107/S1600536810013772

**Published:** 2010-04-21

**Authors:** Fang Ge, Wanli Nie, Maxim V. Borzov, Andrei V. Churakov

**Affiliations:** a Key Laboratory of Synthetic and Natural Chemistry of the Ministry of Education, College of Chemistry and Material Science, The North-West University of Xi’an, Taibai Bei Avenue 229, Xi’an 710069, Shaanxi Province, People’s Republic of China; b Key State Key Laboratory of Elementoorganic Chemistry, Nankai University, Weijing Rd 94, Tianjing 300071, People’s Republic of China; c N. S. Kurnakov Institute of General and Inorganic Chemistry, Russian Academy of Science, Leninskii Prosp. 31, Moscow 119991, Russian Federation

## Abstract

The title compound, [Ti(C_15_H_23_N_2_Si)Cl_3_]·0.5C_4_H_8_O, has been prepared from {2-[2-(η^5^-cyclo­penta­dien­yl)-2-methyl­prop­yl]-1*H*-imidazolyl-κ*N* ^1^}bis­(*N*,*N*-diethyl­amido-κ*N*)titanium(IV), (C_12_H_14_N_2_)Ti(NEt_2_)_2_, by reaction with excess of Me_3_SiCl in tetra­hydro­furan (THF) at 353 K. The crystal structure contains THF as adduct solvent, disordered around a center of inversion. The presence of THF and the adduct ratio has been independently supported by ^1^H NMR spectroscopy. The coordination polyhedron of the Ti atom is distorted square-pyramidal, assuming the cyclo­penta­dienyl (Cp) ring occupies one coordination site. The Ti, Si and CH_2_ group C atoms only deviate slightly from the imidazole ring plane [by 0.021 (4), 0.133 (4) and 0.094 (4) Å, respectively]. Comparison of the principal geometric parameters with those of the few known structurally characterized analogues reveal small differences in bond lengths and angles at the Ti atom. The title complex is only stable in THF-*d*_8_ in the presence of excess Me_3_SiCl, otherwise it exists in an equilibrium with equimolar amounts of dichlorido{2-[2-(η^5^-cyclo­penta­dien­yl)-2-methyl­prop­yl]-1*H*-imidazolyl-κ*N* ^3^}titanium(IV) and chloro­trimethyl­silane.

## Related literature

For a description of cyclo­penta­dienes with pendant 1*H*-imidazol(in)-2-yl side-chain functional groups and group 4 transition metal complexes of general type [η^5^-Cp-(CPh_2_CH_2_)-imidazol(in)e)-κ*N* ^3^]-*M* ^IV^Cl_3_ (*M* = Ti, Zr) , see: Krut’ko *et al.* (2006 ▶); Nie *et al.* (2008 ▶). For the geometric parameters of structurally characterized Ti^IV^ complexes of the similar η^5^-CpTiCl_3_-N*R_n_* type, see: trichloro­{2-[2-(η^5^-cyclo­penta­dien­yl)-2,2-diphenyl­ethyl]-1-methyl-1*H*-imidazole-κ*N* ^3^}titanium(IV), C_23_H_21_Cl_3_N_2_Ti (Krut’ko *et al.*, 2006 ▶); trichloro­{1-[2-(η^5^-cyclo­penta­dien­yl)eth­yl]pyrrolidine-κ*N*}titanium(IV), C_11_H_16_Cl_3_NTi (Herrmann *et al.*, 1995 ▶); trichloro­[8-(η^5^-2,3,4,5-tetra­methyl­cyclo­penta­dien­yl)quinoline-κ*N*]titanium(IV), C_18_H_18_Cl_3_NTi (Enders *et al.*, 1997 ▶); trichloro­[8-(η^5^-2,3-dimethyl­cyclo­penta­dien­yl)quinoline-κ*N*]titanium(IV), C_16_H_14_Cl_3_NTi (Enders *et al.*, 1996 ▶). For the preparation of [2-[2-(η^5^-cyclo­penta­dien­yl)-2-methyl­prop­yl]-1*H*-imidazolyl-κ*N* ^1^]bis­(*N*,*N*-di­ethyl­amido-κ*N*)titanium(IV), (C_12_H_14_N_2_)Ti(NEt_2_)_2_, see: Wang *et al.* (2009 ▶). For a description of the Cambridge Structural Database, see: Allen (2002 ▶).
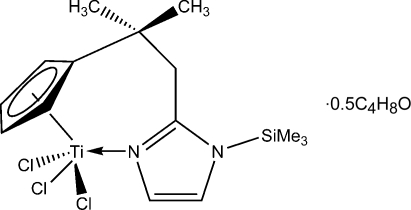


## Experimental

### 

#### Crystal data

[Ti(C_15_H_23_N_2_Si)Cl_3_]·0.5C_4_H_8_O
*M* *_r_* = 449.75
Monoclinic, 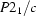

*a* = 8.8033 (9) Å
*b* = 11.8201 (11) Å
*c* = 21.481 (2) Å
β = 99.399 (1)°
*V* = 2205.2 (4) Å^3^
*Z* = 4
Mo *K*α radiation
μ = 0.81 mm^−1^
*T* = 293 K
0.29 × 0.21 × 0.14 mm

#### Data collection

Bruker SMART APEXII diffractometer
Absorption correction: multi-scan (*SADABS*; Sheldrick, 1996 ▶) *T*_min_ = 0.798, *T*_max_ = 0.894
10736 measured reflections
3869 independent reflections
2812 reflections with *I* > 2σ(*I*)
*R*_int_ = 0.031

#### Refinement

*R*[*F* ^2^ > 2σ(*F* ^2^)] = 0.040
*wR*(*F* ^2^) = 0.115
*S* = 1.02
3869 reflections
249 parameters
70 restraints
H-atom parameters constrained
Δρ_max_ = 0.42 e Å^−3^
Δρ_min_ = −0.27 e Å^−3^

### 

Data collection: *APEX2* (Bruker, 2007 ▶); cell refinement: *SAINT* (Bruker, 2007 ▶); data reduction: *SAINT*; program(s) used to solve structure: *SHELXS97* (Sheldrick, 2008 ▶); program(s) used to refine structure: *SHELXL97* (Sheldrick, 2008 ▶); molecular graphics: *SHELXTL* (Sheldrick, 2008 ▶) and *OLEX2* (Dolomanov *et al.*, 2009 ▶); software used to prepare material for publication: *SHELXTL*, *OLEX2* and *PLATON* (Spek, 2009 ▶).

## Supplementary Material

Crystal structure: contains datablocks I, global. DOI: 10.1107/S1600536810013772/nc2182sup1.cif

Structure factors: contains datablocks I. DOI: 10.1107/S1600536810013772/nc2182Isup2.hkl

Additional supplementary materials:  crystallographic information; 3D view; checkCIF report

## Figures and Tables

**Table 1:** Geometrical parameters (Å, °) of the environment of the Ti atom in the title compound compared with those of related structures

	(I)	(III)	(IV)	(V)	(VI)
Ti1—N2	2.153 (2)	2.163 (2)	2.357 (1)	2.274 (4)	2.261 (2)
Ti1—Cl1	2.3475 (9)	2.3513 (8)	2.3217 (4)	2.322 (2)	2.331 (1)
Ti1—Cl2	2.3377 (10)	2.3486 (8)	2.3729 (5)	2.326 (2)	2.338 (1)
Ti1—Cl3	2.3533 (9)	2.3340 (8)	2.2895 (5)	2.300 (2)	2.307 (1)
Ti1⋯Cp_cent_	2.030 (1)	2.036	2.025	2.035	2.047
Ti1⋯*PL*1	2.029 (1)	2.034 (1)	2.025	2.034	2.046
Ti1⋯*PL*2	0.022 (5)	0.608		0.175	0.215
N2—Ti1⋯Cp_cent_	111.2 (1)*^*a*^*	110.20	99.66	101.44	101.64
Cl1—Ti1⋯Cp_cent_	109.2 (1)*^*a*^*	110.08	116.37	116.68	113.90
Cl2—Ti1⋯Cp_cent_	110.69 (9)*^*a*^*	109.75	107.63	109.28	109.73
Cl3—Ti1⋯Cp_cent_	110.58 (9)*^*a*^*	110.93	114.71	113.76	115.56
Cl1—Ti1—N2	80.79 (7)	79.24 (6)	82.57 (2)	78.64	80.32
Cl2—Ti1—N2	138.10 (6)	140.05 (6)	152.70 (3)	149.27	148.63
Cl3—Ti1—N2	80.82 (7)	81.19 (6)	83.45 (2)	80.23	78.94
Cl2—Ti1—Cl1	85.38 (4)	86.06 (3)	85.18 (2)	87.84	87.14
Cl2—Ti1—Cl3	85.07 (4)	86.01 (3)	85.30 (2)	86.95	87.20
Cl3—Ti1—Cl1	139.98 (4)	138.52 (3)	128.55 (2)	128.07	129.12
*PL*1–*PL*2	81.0 (1)	78.335		82.491	85.895

Notes: (*a*) the angle between the Ti1—N2 bond and the normal to *PL*1; (I) this work; (III) Krut’ko *et al.* (2006 ▶); (IV) Herrmann *et al.* (1995 ▶); (V) Enders *et al.* (1997 ▶); (VI) Enders *et al.* (1996 ▶). *PL*1 and Cp_cent_ denote the C11–C15 Cp ring r.m.s. plane and centroid, respectively, while *PL*2 denotes an r.m.s. plane through the non-H atoms of a heterocyclic ring.

**Fig. 1.**
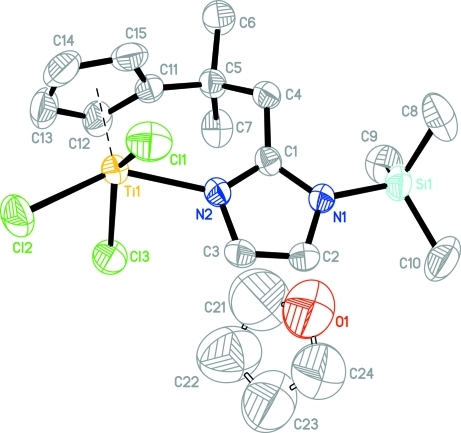
The asymmetric unit of the title compound (I) with labelling and thermal ellipsoids drawn at the 50% probability level. H-atoms are omitted for clarity.

**Fig. 2.**
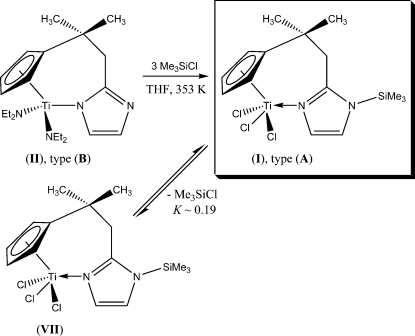
The formation of the title compound.

## supplementary crystallographic information

### Comment

Cyclopentadienes (Cp-s) with pendant 1*H*-imidazol(in)-2-yl side-chain functional groups and the Group 4 transition metal complexes of general type [η^5^-Cp-(C_2_-link)-imidazol(in)e)-κ*N*^3^]-M^IV^Cl_3_ (*M* = Ti, Zr), (A), based on them were described not long ago (Krut'ko *et al.*, 2006; Nie *et al.*, 2008). All Ti and Zr complexes reported in these papers possess Me-groups at the N1 atoms and were prepared by reactions of metal tetrachlorides with monolithium- or trimethylsilyl-derivatives of parent cyclopentadienes. Recently a synthetic approach to η^5^-Cp-tris(*sec*-amido)Ti^IV^ type complexes of general formula [η^5^-Cp-(C_1 or 2_-link)-imidazolyl-κ*N*^1^]-M^IV^(NEt_2_)_2_, (B), was suggested (Wang *et al.*, 2009). Here we report on the crystal structure of trichloro{2-[2-(η^5^-cyclopentadienyl)-2-methylpropyl]-1-trimethylsilyl-1*H*-imidazole-κ*N*^3^}titanium(IV) that crystallizes as an 1:0.5 adduct with tetrahydrofuran (THF) (I), which was prepared by a reaction with chlorotrimethylsilane via a facile (B) to (A) conversion method. The molecular structure of (I) is discussed in comparison with those of its few known analogues.

Complex (I) was prepared by treatment of [2-[2-(η^5^-cyclopentadienyl)-2-methylpropyl]-1*H*-imidazolyl-κ*N*^1^]bis(*N*,*N*-diethylamido-κ*N*)titanium(IV), (C_12_H_14_N_2_)Ti(NEt_2_)_2_, (II), with excess of Me_3_SiCl in a THF medium at elevated temperature (see Fig. 2 and Experimental for details). The presence of THF in the crystal and the adduct ratio were not evident in the structure solution and refinement stages but were independently supported by ^1^H NMR spectroscopy data [multiplets at δ(H) 1.78 and 3.62 p.p.m. with both of the relative integral intensities corresponding to 2 H]. The THF molecule was found and located using SQUEEZE in the *PLATON* program package (Spek, 2009) which retrieved two voids at (1/2, 0, 1/2) and (1/2, 1/2, 1) (each of 180 Å^3^ and 42ē; total electron count per unit cell 84ē). In the final structure model, the adduct solvent molecule was treated as disordered around a center of inversion (1/2, 0, 1/2) (see Refinement section for details).

An analysis in the Cambridge Structural Database (CSD; Version 5.27, release February 2009; Allen, 2002) reveals only 4 structurally characterized Ti^IV^ complexes of the similar η^5^-CpTiCl_3_-NR_n_ type (4 independent fragments): trichloro{2-[2-(η^5^-cyclopentadienyl)-2,2-diphenylethyl]-1-methyl-1*H*-imidazole-κ*N*^3^}titanium(IV), C_23_H_21_Cl_3_N_2_Ti, (III), (Krut'ko *et al.*, 2006); trichloro{1-[2-(η^5^-cyclopentadienyl)ethyl]pyrrolidine-κ*N*}titanium(IV), C_11_H_16_Cl_3_NTi, (IV), (Herrmann *et al.*, 1995); trichloro[8-(η^5^-2,3,4,5-tetramethylcyclopentadienyl)quinoline-κ*N*]titanium(IV), C_18_H_18_Cl_3_NTi, (V), (Enders *et al.*, 1997); and trichloro[8-(η^5^-2,3-dimethylcyclopentadienyl)quinoline-κ*N*]titanium(IV), C_16_H_14_Cl_3_NTi, (VI), (Enders *et al.*, 1996).

All complexes of question exhibit one and the same structural motif. They are mononuclear complexes, with the coordination environment of the Ti-atoms being a distorted square pyramid (assuming Cp-rings occupy one coordination site; "four-leg piano stool"). Contents of the unit cells are presented by pairs of enantiomorphic conformers connected by inversion symmetry operations. In all complexes under discussion, ligating N-atoms are linked to Cp-groups with a C_2_ [(IV)-(VI)] or C_3_ [(I) and (III)] bridges. Noteworthy, that no structurally characterized complexes of type η^5^-CpTiCl_3_-NR_n_ with a non-linked to Cp NR_n_ functionality are known at the moment.

Compounds (I) and (III) represent a pair of the "closest relatives", and, despite of the evident differences in their chemical structure (CPh_2_ against CMe_2_ and NMe against NSiMe_3_), the geometrical parameters of the coordination environment of the Ti-atoms and imidazole rings nearly match (see Table 1). This is the same for the torsion angles in the bridge [C4—C5—C11—C12 and C1—C4—C5—C11 in (I) and the related angles in (III); compare –136.8 (3) and 64.3 (3)° in (I) with –135.21 (4) and 61.22 (5)° in (III)]. However, while in the main molecule of (I), the Ti-, Si- and CH_2_-group carbon atoms deviate only slightly from the imidazole ring r. m. s. plane [PL2; by 0.022 (5), 0.133 (4) and 0.094 (5) Å, respectively], the Ti-atom in (III) noticeably deviates from the imidazole r. m. s. plane (by 0.608 Å) what could be, at the first glance, explained by a mutual repulsion of the spatially adjacent imidazole and phenyl rings. Another difference in the crystal structures of (I) and (III) is due to the presence of a bulky SiMe_3_ group in (I). These groups are stretched outwards of the main molecule and "pump up" the unit cell volume [compare *V* = 2205.2 (4) Å^3^ in (I) with 2098.8, 1304.8, 1567.9, and 1749.9 Å^3^ in (III)-(VI), respectively] what causes appearance of voids suitable for THF molecules.

Elongation of the bridge from C_2_ [in (IV)-(VI)] to C_3_ [in (I) and (III)] has a little effect on the Ti1—PL1 (or Cp_cent_; PL1 and Cp_cent_ denote r.m.s. plane and centroid of the Cp-ring, respectively) distances, as well as on the angle Cl2–Ti1–Cp_cent_ and "*cis*-angles" Cl2–Ti1–Cl1, Cl2–Ti1–Cl3, Cl1–Ti1–N2, and Cl3–Ti1–N2. However, the angles N2–Ti1–Cp_cent_ in (I) and (III) are expanded by approximately 10° compared to those in (IV)-(VI) while the "*trans*-angle" Cl2–Ti1–N2 is tightened by the same value. The angles Cl1–Ti1–Cp_cent_ and Cl3–Ti1–Cp_cent_ in (I) and (III) are decreased by approximately 5° comparatively to those in (IV)-(VI) while the "*trans*-angle" Cl1–Ti1–Cl3 is increased by approximately 10°.

### Experimental

All operations were performed in all-sealed evacuated glass vessels with application of the high-vacuum line (the residual pressure of non-condensible gases within 1.5–1.0×10 ^-3^ Torr range, 1 Torr = 133.322 Pa). Complex (II) was prepared as described in our earlier work (Wang *et al.*, 2009). THF and THF-*d*_8_ were kept with disodium benzophenone ketyl and transferred into reaction vessels and/or NMR tubes on the high-vacuum line by trapping the vapour with liq. N_2_. Chlorotrimethylsilane was refluxed with and kept over CaH_2_ and transferred into reaction vessels in a similar way. — NMR spectra were recorded on Varian INOVA-400 instrument. For ^13^C{^1^H}and ^1^H NMR spectra, the ^13^C and residual proton resonance of the *d*-solvent [δ_H_ = 1.73 and δ_C_ = 25.3 (THF-*d*_8_)] were used as internal reference standards.

Complex (I): To a solution of (II) (0.282 g, 0.75 mmol) in THF (20 ml), an excess of Me_3_SiCl (0.6 ml, 4.71 mmol) was added at approx. 253 K. An immediate precipitation of a yellow fine-crystalline solid occurred. The reaction mixture was then heated at 353 K until all the solid dissolved, the volume was reduced two times and the mother liquor was allowed to cool gradually along with the water bath down to ambient temperature. On the walls of the reaction vessel well formed bright-orange crystals grew. The orange mother liquor was removed from the crystals by decantation, the solid was rinsed once with cold (253 K) THF and the crystals were quickly dried by trapping all volatiles with liquid N_2_. Yield 0.275 g (82%). — ^1^H NMR (THF-*d*_8_, 296 K): δ = 0.58 [s, 9 H, Si(C*H*_3_)_3_], 1.30 [s, 6 H, C(C*H*_3_)_2_], 1.78 (m, 2 H, 3- and 4-C*H*_2_ in THF), 3.25 (s, CH_2_), 3.62 (broadened m, 2 H, 2- and 5-C*H*_2_ in THF), 6.91 (broadened m, 4 H, C_5_*H*_4_), 6.95, 7.52 (both broadened d, 1 H + 1 H, ^3^*J*_HH_ = 2.3 Hz, C*H* in imidazole). — ^13^C{^1^H} NMR (THF-*d*_8_, 296 K): δ = 0.09 [Si(*C*H_3_)_3_], 28.81 (C(*C*H_3_)_2_), 36.11 (CH_2_), 41.72 [*C*(CH_3_)_2_], 119.99, 131.05 (*C*H in imidazole), 122.71, 124.49 (*C*H in C_5_H_4_, double intensity), 145.72 (*C* in C_5_H_4_), 149.73 (*C* in imidazole). — Admixture of (VII): ^1^H NMR (THF-*d*_8_, 296 K): δ = 1.26 [s, 6 H, C(C*H*_3_)_2_], 3.15 (s, CH_2_), 6.87, 6.92 (both broadened virt. t, 4 H, ^3 + 4^*J*_HH_ =2.7 Hz, C_5_*H*_4_), 6.96, 7.48 (both broadened unresolved d, 1 H + 1 H, C*H* in imidazole). — Admixture of Me_3_SiCl: ^1^H NMR (THF-*d*_8_, 296 K): δ = 0.41 [s, 9 H, Si(C*H*_3_)_3_]. — ^13^C{^1^H} NMR (THF-*d*_8_, 296 K): δ = 3.10 [Si(*C*H_3_)_3_]. Content of complex (VII) and Me_3_SiCl in the reaction mixture is 16% (mol.) Low concentration of (VII) made its signals in ^13^C{^1^H} NMR spectrum of the equilibrium mixture invisible.

Single crystal of I suitable for X-ray diffraction analysis were picked up directly from the isolated materials (N_2_-filled glove-box) and mounted inside a Lindemann glass capillary (diameter 0.5 mm).

### Refinement

The non-H atoms were refined anisotropically. The H atoms were treated as riding atoms with distances C—H = 0.96 (CH_3_), 0.97 (CH_2_), 0.93 Å (C_Ar_H), and *U*_iso_(H) = 1.5 *U*_eq_(C), 1.2 *U*_eq_(C), and 1.2 *U*_eq_(C), respectively. The THF molecule is disordered around inversion center (1/2, 0, 1/2) and was treated with a "PART -1" instruction and *sof* constrained to 0.5. O—C, (O)C—C, C—C 1,2-distances and corresponding 1,3-distances were restrained to 1.421 (6), 1.482 (6), 1.498 (6) and 2.329 (10), 2.344 (10), 2.302 (10) Å, respectively [DFIX instructions; distance values and their standard uncertainties (su-s) were chosen on the basis of the statistical analysis of the CSD (Version 5.27, release February 2009; Allen, 2002) for non-disordered solvent THF molecules (*R*_max_ = 5.0; 70 hits and 99 fragments; 61 fragments used for statistical analysis on rejecting hits with pathological fragments)]. Non-hydrogen atoms of the disordered THF molecule were restrained to behave approximately isotropically with su 0.01 Å^2^ (ISOR instruction). The anisotropic displacement parameters (ADP-s) for these atoms were restrained to be the same with su of 0.01 Å^2^ (SIMU instruction).

### Crystal data

**Table:**

[Ti(C_15_H_23_N_2_Si)Cl_3_]·0.5C_4_H_8_O	*F*(000) = 936
*M**_r_* = 449.75	*D*_x_ = 1.355 Mg m^−^^3^
Monoclinic, *P*2_1_/*c*	Mo *K*α radiation, λ = 0.71073 Å
Hall symbol: -P 2ybc	Cell parameters from 2833 reflections
*a* = 8.8033 (9) Å	θ = 2.6–24.1°
*b* = 11.8201 (11) Å	µ = 0.81 mm^−^^1^
*c* = 21.481 (2) Å	*T* = 293 K
β = 99.399 (1)°	Block, orange
*V* = 2205.2 (4) Å^3^	0.29 × 0.21 × 0.14 mm
*Z* = 4	

### Data collection

**Table:**

Bruker SMART APEXII diffractometer	3869 independent reflections
Radiation source: fine-focus sealed tube	2812 reflections with *I* > 2σ(*I*)
graphite	*R*_int_ = 0.031
Detector resolution: 8.333 pixels mm^-1^	θ_max_ = 25.0°, θ_min_ = 1.9°
phi and ω scans	*h* = −10→6
Absorption correction: multi-scan (*SADABS*; Sheldrick, 1996)	*k* = −14→14
*T*_min_ = 0.798, *T*_max_ = 0.894	*l* = −24→25
10736 measured reflections	

### Refinement

**Table:**

Refinement on *F*^2^	Primary atom site location: structure-invariant direct methods
Least-squares matrix: full	Secondary atom site location: difference Fourier map
*R*[*F*^2^ > 2σ(*F*^2^)] = 0.040	Hydrogen site location: inferred from neighbouring sites
*wR*(*F*^2^) = 0.115	H-atom parameters constrained
*S* = 1.02	*w* = 1/[σ^2^(*F*_o_^2^) + (0.0702*P*)^2^] where *P* = (*F*_o_^2^ + 2*F*_c_^2^)/3
3869 reflections	(Δ/σ)_max_ < 0.001
249 parameters	Δρ_max_ = 0.42 e Å^−^^3^
70 restraints	Δρ_min_ = −0.27 e Å^−^^3^

### Special details

**Table:**

Geometry. All esds (except the esd in the dihedral angle between two l.s. planes) are estimated using the full covariance matrix. The cell esds are taken into account individually in the estimation of esds in distances, angles and torsion angles; correlations between esds in cell parameters are only used when they are defined by crystal symmetry. An approximate (isotropic) treatment of cell esds is used for estimating esds involving l.s. planes.
Refinement. Refinement of *F*^2^ against ALL reflections. The weighted *R*-factor wR and goodness of fit *S* are based on *F*^2^, conventional *R*-factors *R* are based on *F*, with *F* set to zero for negative *F*^2^. The threshold expression of *F*^2^ > σ(*F*^2^) is used only for calculating *R*-factors(gt) etc. and is not relevant to the choice of reflections for refinement. *R*-factors based on *F*^2^ are statistically about twice as large as those based on *F*, and *R*- factors based on ALL data will be even larger.

### Fractional atomic coordinates and isotropic or equivalent isotropic displacement parameters (Å^2^)

**Table:**

	*x*	*y*	*z*	*U*_iso_*/*U*_eq_	Occ. (<1)
Ti1	0.43495 (6)	0.55311 (4)	0.32607 (2)	0.03818 (17)	
Cl1	0.38088 (10)	0.37906 (7)	0.37034 (4)	0.0627 (3)	
Cl2	0.67102 (10)	0.47125 (8)	0.31535 (5)	0.0702 (3)	
Cl3	0.57958 (10)	0.71852 (7)	0.35301 (4)	0.0566 (2)	
Si1	0.00773 (10)	0.73534 (8)	0.49770 (4)	0.0542 (3)	
N1	0.1676 (2)	0.67619 (19)	0.46582 (10)	0.0396 (5)	
N2	0.3202 (2)	0.61390 (19)	0.40069 (10)	0.0391 (5)	
C1	0.1772 (3)	0.6447 (2)	0.40551 (12)	0.0352 (6)	
C2	0.3137 (3)	0.6600 (3)	0.50049 (13)	0.0491 (8)	
H2	0.3426	0.6725	0.5435	0.059*	
C3	0.4050 (3)	0.6234 (3)	0.46088 (13)	0.0487 (8)	
H3	0.5092	0.6069	0.4719	0.058*	
C4	0.0459 (3)	0.6406 (2)	0.35326 (12)	0.0401 (7)	
H4B	−0.0408	0.6794	0.3663	0.048*	
H4A	0.0164	0.5623	0.3451	0.048*	
C5	0.0790 (3)	0.6941 (3)	0.29174 (13)	0.0424 (7)	
C6	−0.0695 (4)	0.6866 (3)	0.24248 (14)	0.0637 (10)	
H6B	−0.0565	0.7290	0.2056	0.096*	
H6C	−0.1542	0.7172	0.2601	0.096*	
H6A	−0.0902	0.6089	0.2311	0.096*	
C7	0.1269 (4)	0.8181 (3)	0.30352 (16)	0.0567 (8)	
H7B	0.2190	0.8216	0.3343	0.085*	
H7C	0.0460	0.8586	0.3188	0.085*	
H7A	0.1457	0.8517	0.2648	0.085*	
C8	−0.1419 (4)	0.6251 (4)	0.4950 (2)	0.0828 (12)	
H8A	−0.1668	0.5961	0.4528	0.124*	
H8C	−0.2325	0.6572	0.5075	0.124*	
H8B	−0.1043	0.5648	0.5232	0.124*	
C9	−0.0605 (5)	0.8622 (3)	0.45119 (18)	0.0730 (11)	
H9B	0.0263	0.9042	0.4418	0.110*	
H9C	−0.1193	0.9089	0.4751	0.110*	
H9A	−0.1241	0.8396	0.4126	0.110*	
C10	0.0866 (5)	0.7784 (5)	0.57934 (18)	0.1059 (17)	
H10A	0.1331	0.7143	0.6024	0.159*	
H10C	0.0050	0.8071	0.5996	0.159*	
H10B	0.1627	0.8364	0.5785	0.159*	
C11	0.2036 (3)	0.6279 (3)	0.26614 (12)	0.0424 (7)	
C12	0.3285 (4)	0.6727 (3)	0.24110 (13)	0.0528 (8)	
H12	0.3546	0.7489	0.2402	0.063*	
C13	0.4073 (4)	0.5834 (4)	0.21778 (15)	0.0704 (11)	
H13	0.4951	0.5897	0.1991	0.084*	
C14	0.3307 (5)	0.4838 (4)	0.22756 (16)	0.0705 (11)	
H14	0.3578	0.4115	0.2162	0.085*	
C15	0.2056 (4)	0.5111 (3)	0.25741 (14)	0.0535 (8)	
H15	0.1356	0.4599	0.2694	0.064*	
O1	0.3332 (13)	0.9741 (12)	0.4938 (7)	0.206 (4)	0.50
C21	0.3746 (18)	1.0279 (16)	0.4404 (6)	0.205 (5)	0.50
H21B	0.3099	1.0935	0.4290	0.246*	0.50
H21A	0.3621	0.9763	0.4048	0.246*	0.50
C22	0.5356 (19)	1.0621 (16)	0.4569 (7)	0.199 (5)	0.50
H22B	0.5489	1.1392	0.4433	0.239*	0.50
H22A	0.6009	1.0129	0.4367	0.239*	0.50
C23	0.5763 (17)	1.0534 (16)	0.5268 (8)	0.201 (5)	0.50
H23A	0.6671	1.0067	0.5385	0.241*	0.50
H23B	0.5964	1.1277	0.5455	0.241*	0.50
C24	0.443 (2)	1.0019 (16)	0.5477 (6)	0.195 (5)	0.50
H24A	0.4741	0.9342	0.5720	0.234*	0.50
H24B	0.3984	1.0542	0.5744	0.234*	0.50

### Atomic displacement parameters (Å^2^)

**Table:**

	*U*^11^	*U*^22^	*U*^33^	*U*^12^	*U*^13^	*U*^23^
Ti1	0.0335 (3)	0.0417 (3)	0.0402 (3)	0.0018 (2)	0.0084 (2)	−0.0042 (2)
Cl1	0.0673 (6)	0.0486 (5)	0.0696 (6)	−0.0078 (4)	0.0033 (4)	0.0064 (4)
Cl2	0.0459 (5)	0.0750 (6)	0.0929 (7)	0.0117 (4)	0.0209 (4)	−0.0205 (5)
Cl3	0.0591 (5)	0.0518 (5)	0.0609 (5)	−0.0144 (4)	0.0157 (4)	−0.0023 (4)
Si1	0.0444 (5)	0.0776 (6)	0.0435 (5)	0.0092 (5)	0.0156 (4)	−0.0045 (4)
N1	0.0353 (13)	0.0528 (14)	0.0313 (12)	0.0003 (11)	0.0075 (10)	0.0005 (10)
N2	0.0299 (12)	0.0521 (14)	0.0358 (12)	0.0034 (11)	0.0065 (10)	−0.0004 (10)
C1	0.0330 (15)	0.0366 (14)	0.0373 (15)	0.0003 (11)	0.0092 (11)	0.0052 (11)
C2	0.0371 (17)	0.072 (2)	0.0363 (16)	0.0007 (15)	0.0012 (13)	−0.0022 (15)
C3	0.0305 (16)	0.074 (2)	0.0390 (16)	0.0047 (15)	−0.0018 (13)	0.0020 (15)
C4	0.0325 (15)	0.0500 (17)	0.0373 (15)	−0.0022 (13)	0.0041 (12)	0.0015 (13)
C5	0.0356 (16)	0.0548 (18)	0.0367 (15)	0.0058 (13)	0.0061 (12)	0.0057 (13)
C6	0.0438 (18)	0.105 (3)	0.0392 (17)	0.0132 (19)	−0.0028 (14)	0.0103 (17)
C7	0.066 (2)	0.0485 (18)	0.059 (2)	0.0106 (16)	0.0195 (17)	0.0141 (15)
C8	0.054 (2)	0.104 (3)	0.098 (3)	0.003 (2)	0.033 (2)	0.024 (2)
C9	0.070 (2)	0.064 (2)	0.086 (3)	0.0130 (19)	0.016 (2)	−0.011 (2)
C10	0.094 (3)	0.174 (5)	0.052 (2)	0.028 (3)	0.019 (2)	−0.029 (3)
C11	0.0385 (16)	0.0572 (18)	0.0300 (14)	0.0030 (14)	0.0013 (12)	0.0018 (13)
C12	0.054 (2)	0.069 (2)	0.0370 (16)	0.0061 (17)	0.0123 (14)	0.0125 (15)
C13	0.062 (2)	0.112 (3)	0.0396 (18)	0.021 (2)	0.0170 (17)	−0.002 (2)
C14	0.075 (3)	0.083 (3)	0.049 (2)	0.020 (2)	−0.0031 (18)	−0.0266 (19)
C15	0.0491 (19)	0.059 (2)	0.0475 (17)	0.0001 (16)	−0.0070 (15)	−0.0126 (15)
O1	0.195 (6)	0.228 (7)	0.195 (6)	0.011 (6)	0.036 (5)	−0.014 (6)
C21	0.199 (7)	0.212 (7)	0.203 (7)	−0.002 (6)	0.031 (6)	0.017 (6)
C22	0.203 (7)	0.199 (8)	0.193 (7)	−0.014 (6)	0.024 (6)	0.008 (6)
C23	0.206 (7)	0.199 (7)	0.199 (7)	−0.014 (6)	0.039 (6)	−0.005 (6)
C24	0.188 (7)	0.211 (7)	0.187 (7)	−0.007 (6)	0.038 (6)	−0.011 (6)

### Geometric parameters (Å, °)

**Table:**

Ti1—N2	2.153 (2)	C7—H7A	0.9600
Ti1—C14	2.315 (3)	C8—H8A	0.9600
Ti1—C13	2.327 (3)	C8—H8C	0.9600
Ti1—Cl2	2.3377 (10)	C8—H8B	0.9600
Ti1—Cl1	2.3475 (9)	C9—H9B	0.9600
Ti1—C15	2.350 (3)	C9—H9C	0.9600
Ti1—Cl3	2.3533 (9)	C9—H9A	0.9600
Ti1—C12	2.377 (3)	C10—H10A	0.9600
Ti1—C11	2.394 (3)	C10—H10C	0.9600
Si1—N1	1.804 (2)	C10—H10B	0.9600
Si1—C8	1.847 (4)	C11—C15	1.394 (4)
Si1—C9	1.847 (4)	C11—C12	1.404 (4)
Si1—C10	1.849 (4)	C12—C13	1.399 (5)
N1—C1	1.364 (3)	C12—H12	0.9300
N1—C2	1.390 (3)	C13—C14	1.390 (5)
N2—C1	1.330 (3)	C13—H13	0.9300
N2—C3	1.388 (3)	C14—C15	1.399 (5)
C1—C4	1.474 (4)	C14—H14	0.9300
C2—C3	1.335 (4)	C15—H15	0.9300
C2—H2	0.9300	O1—C21	1.410 (6)
C3—H3	0.9300	O1—C24	1.421 (6)
C4—C5	1.535 (4)	C21—C22	1.460 (6)
C4—H4B	0.9700	C21—H21B	0.9700
C4—H4A	0.9700	C21—H21A	0.9700
C5—C11	1.522 (4)	C22—C23	1.489 (6)
C5—C7	1.534 (4)	C22—H22B	0.9700
C5—C6	1.544 (4)	C22—H22A	0.9700
C6—H6B	0.9600	C23—C24	1.456 (6)
C6—H6C	0.9600	C23—H23A	0.9700
C6—H6A	0.9600	C23—H23B	0.9700
C7—H7B	0.9600	C24—H24A	0.9700
C7—H7C	0.9600	C24—H24B	0.9700
			
N2—Ti1—C14	129.38 (12)	H7B—C7—H7C	109.5
N2—Ti1—C13	135.04 (11)	C5—C7—H7A	109.5
C14—Ti1—C13	34.86 (13)	H7B—C7—H7A	109.5
N2—Ti1—Cl2	138.10 (6)	H7C—C7—H7A	109.5
C14—Ti1—Cl2	89.41 (10)	Si1—C8—H8A	109.5
C13—Ti1—Cl2	85.12 (9)	Si1—C8—H8C	109.5
N2—Ti1—Cl1	80.79 (7)	H8A—C8—H8C	109.5
C14—Ti1—Cl1	89.07 (11)	Si1—C8—H8B	109.5
C13—Ti1—Cl1	123.02 (12)	H8A—C8—H8B	109.5
Cl2—Ti1—Cl1	85.38 (4)	H8C—C8—H8B	109.5
N2—Ti1—C15	94.48 (10)	Si1—C9—H9B	109.5
C14—Ti1—C15	34.89 (12)	Si1—C9—H9C	109.5
C13—Ti1—C15	57.74 (14)	H9B—C9—H9C	109.5
Cl2—Ti1—C15	122.49 (8)	Si1—C9—H9A	109.5
Cl1—Ti1—C15	81.89 (9)	H9B—C9—H9A	109.5
N2—Ti1—Cl3	80.82 (7)	H9C—C9—H9A	109.5
C14—Ti1—Cl3	129.55 (12)	Si1—C10—H10A	109.5
C13—Ti1—Cl3	94.70 (12)	Si1—C10—H10C	109.5
Cl2—Ti1—Cl3	85.07 (4)	H10A—C10—H10C	109.5
Cl1—Ti1—Cl3	139.98 (4)	Si1—C10—H10B	109.5
C15—Ti1—Cl3	134.78 (9)	H10A—C10—H10B	109.5
N2—Ti1—C12	101.49 (10)	H10C—C10—H10B	109.5
C14—Ti1—C12	57.36 (13)	C15—C11—C12	107.1 (3)
C13—Ti1—C12	34.59 (12)	C15—C11—C5	125.7 (3)
Cl2—Ti1—C12	114.57 (8)	C12—C11—C5	126.9 (3)
Cl1—Ti1—C12	138.74 (9)	C15—C11—Ti1	71.21 (16)
C15—Ti1—C12	56.85 (12)	C12—C11—Ti1	72.22 (17)
Cl3—Ti1—C12	79.88 (9)	C5—C11—Ti1	126.58 (18)
N2—Ti1—C11	79.37 (9)	C13—C12—C11	108.5 (3)
C14—Ti1—C11	57.56 (11)	C13—C12—Ti1	70.76 (19)
C13—Ti1—C11	57.62 (11)	C11—C12—Ti1	73.55 (16)
Cl2—Ti1—C11	142.36 (7)	C13—C12—H12	125.7
Cl1—Ti1—C11	109.31 (8)	C11—C12—H12	125.7
C15—Ti1—C11	34.16 (10)	Ti1—C12—H12	121.6
Cl3—Ti1—C11	101.76 (8)	C14—C13—C12	107.7 (3)
C12—Ti1—C11	34.23 (10)	C14—C13—Ti1	72.1 (2)
N1—Si1—C8	108.14 (16)	C12—C13—Ti1	74.65 (18)
N1—Si1—C9	108.33 (15)	C14—C13—H13	126.2
C8—Si1—C9	112.84 (18)	C12—C13—H13	126.2
N1—Si1—C10	105.75 (16)	Ti1—C13—H13	119.0
C8—Si1—C10	112.3 (2)	C13—C14—C15	108.1 (3)
C9—Si1—C10	109.1 (2)	C13—C14—Ti1	73.0 (2)
C1—N1—C2	106.0 (2)	C15—C14—Ti1	73.94 (17)
C1—N1—Si1	129.77 (19)	C13—C14—H14	125.9
C2—N1—Si1	124.11 (19)	C15—C14—H14	125.9
C1—N2—C3	106.1 (2)	Ti1—C14—H14	118.9
C1—N2—Ti1	135.58 (18)	C11—C15—C14	108.6 (3)
C3—N2—Ti1	118.30 (18)	C11—C15—Ti1	74.63 (16)
N2—C1—N1	110.8 (2)	C14—C15—Ti1	71.16 (19)
N2—C1—C4	124.5 (2)	C11—C15—H15	125.7
N1—C1—C4	124.7 (2)	C14—C15—H15	125.7
C3—C2—N1	107.7 (2)	Ti1—C15—H15	120.2
C3—C2—H2	126.2	C21—O1—C24	109.0 (5)
N1—C2—H2	126.2	O1—C21—C22	107.3 (5)
C2—C3—N2	109.4 (2)	O1—C21—H21B	110.3
C2—C3—H3	125.3	C22—C21—H21B	110.3
N2—C3—H3	125.3	O1—C21—H21A	110.3
C1—C4—C5	114.0 (2)	C22—C21—H21A	110.3
C1—C4—H4B	108.8	H21B—C21—H21A	108.5
C5—C4—H4B	108.8	C21—C22—C23	106.8 (5)
C1—C4—H4A	108.8	C21—C22—H22B	110.4
C5—C4—H4A	108.8	C23—C22—H22B	110.4
H4B—C4—H4A	107.7	C21—C22—H22A	110.4
C11—C5—C7	110.8 (2)	C23—C22—H22A	110.4
C11—C5—C4	110.4 (2)	H22B—C22—H22A	108.6
C7—C5—C4	109.7 (2)	C24—C23—C22	105.4 (5)
C11—C5—C6	107.6 (2)	C24—C23—H23A	110.7
C7—C5—C6	110.3 (3)	C22—C23—H23A	110.7
C4—C5—C6	107.9 (2)	C24—C23—H23B	110.7
C5—C6—H6B	109.5	C22—C23—H23B	110.7
C5—C6—H6C	109.5	H23A—C23—H23B	108.8
H6B—C6—H6C	109.5	O1—C24—C23	108.8 (5)
C5—C6—H6A	109.5	O1—C24—H24A	109.9
H6B—C6—H6A	109.5	C23—C24—H24A	109.9
H6C—C6—H6A	109.5	O1—C24—H24B	109.9
C5—C7—H7B	109.5	C23—C24—H24B	109.9
C5—C7—H7C	109.5	H24A—C24—H24B	108.3
			
C8—Si1—N1—C1	68.5 (3)	N2—Ti1—C12—C13	−167.7 (2)
C9—Si1—N1—C1	−54.1 (3)	C14—Ti1—C12—C13	−38.0 (2)
C10—Si1—N1—C1	−171.0 (3)	Cl2—Ti1—C12—C13	34.1 (3)
C8—Si1—N1—C2	−115.4 (3)	Cl1—Ti1—C12—C13	−78.6 (3)
C9—Si1—N1—C2	121.9 (3)	C15—Ti1—C12—C13	−79.9 (3)
C10—Si1—N1—C2	5.1 (3)	Cl3—Ti1—C12—C13	113.9 (2)
C14—Ti1—N2—C1	−14.3 (3)	C11—Ti1—C12—C13	−117.0 (3)
C13—Ti1—N2—C1	32.8 (3)	N2—Ti1—C12—C11	−50.70 (19)
Cl2—Ti1—N2—C1	−167.8 (2)	C14—Ti1—C12—C11	79.0 (2)
Cl1—Ti1—N2—C1	−95.4 (3)	C13—Ti1—C12—C11	117.0 (3)
C15—Ti1—N2—C1	−14.4 (3)	Cl2—Ti1—C12—C11	151.15 (15)
Cl3—Ti1—N2—C1	120.3 (3)	Cl1—Ti1—C12—C11	38.4 (2)
C12—Ti1—N2—C1	42.7 (3)	C15—Ti1—C12—C11	37.15 (17)
C11—Ti1—N2—C1	16.4 (3)	Cl3—Ti1—C12—C11	−129.07 (18)
C14—Ti1—N2—C3	163.4 (2)	C11—C12—C13—C14	0.7 (4)
C13—Ti1—N2—C3	−149.5 (2)	Ti1—C12—C13—C14	65.0 (2)
Cl2—Ti1—N2—C3	9.9 (3)	C11—C12—C13—Ti1	−64.3 (2)
Cl1—Ti1—N2—C3	82.3 (2)	N2—Ti1—C13—C14	−97.7 (3)
C15—Ti1—N2—C3	163.3 (2)	Cl2—Ti1—C13—C14	96.0 (2)
Cl3—Ti1—N2—C3	−62.0 (2)	Cl1—Ti1—C13—C14	14.7 (3)
C12—Ti1—N2—C3	−139.6 (2)	C15—Ti1—C13—C14	−37.7 (2)
C11—Ti1—N2—C3	−165.9 (2)	Cl3—Ti1—C13—C14	−179.4 (2)
C3—N2—C1—N1	1.5 (3)	C12—Ti1—C13—C14	−114.8 (3)
Ti1—N2—C1—N1	179.43 (18)	C11—Ti1—C13—C14	−78.4 (2)
C3—N2—C1—C4	−176.1 (3)	N2—Ti1—C13—C12	17.2 (3)
Ti1—N2—C1—C4	1.8 (4)	C14—Ti1—C13—C12	114.8 (3)
C2—N1—C1—N2	−2.0 (3)	Cl2—Ti1—C13—C12	−149.2 (2)
Si1—N1—C1—N2	174.59 (19)	Cl1—Ti1—C13—C12	129.5 (2)
C2—N1—C1—C4	175.6 (3)	C15—Ti1—C13—C12	77.1 (2)
Si1—N1—C1—C4	−7.8 (4)	Cl3—Ti1—C13—C12	−64.6 (2)
C1—N1—C2—C3	1.7 (3)	C11—Ti1—C13—C12	36.4 (2)
Si1—N1—C2—C3	−175.2 (2)	C12—C13—C14—C15	−0.6 (4)
N1—C2—C3—N2	−0.8 (4)	Ti1—C13—C14—C15	66.2 (2)
C1—N2—C3—C2	−0.4 (3)	C12—C13—C14—Ti1	−66.7 (2)
Ti1—N2—C3—C2	−178.8 (2)	N2—Ti1—C14—C13	115.0 (2)
N2—C1—C4—C5	−48.5 (4)	Cl2—Ti1—C14—C13	−82.3 (2)
N1—C1—C4—C5	134.2 (3)	Cl1—Ti1—C14—C13	−167.7 (2)
C1—C4—C5—C11	64.3 (3)	C15—Ti1—C14—C13	115.2 (3)
C1—C4—C5—C7	−58.2 (3)	Cl3—Ti1—C14—C13	0.8 (3)
C1—C4—C5—C6	−178.4 (2)	C12—Ti1—C14—C13	37.7 (2)
C7—C5—C11—C15	171.9 (3)	C11—Ti1—C14—C13	78.6 (2)
C4—C5—C11—C15	50.2 (4)	N2—Ti1—C14—C15	−0.2 (3)
C6—C5—C11—C15	−67.4 (4)	C13—Ti1—C14—C15	−115.2 (3)
C7—C5—C11—C12	−15.0 (4)	Cl2—Ti1—C14—C15	162.5 (2)
C4—C5—C11—C12	−136.8 (3)	Cl1—Ti1—C14—C15	77.1 (2)
C6—C5—C11—C12	105.7 (3)	Cl3—Ti1—C14—C15	−114.4 (2)
C7—C5—C11—Ti1	79.7 (3)	C12—Ti1—C14—C15	−77.5 (2)
C4—C5—C11—Ti1	−42.1 (3)	C11—Ti1—C14—C15	−36.6 (2)
C6—C5—C11—Ti1	−159.6 (2)	C12—C11—C15—C14	0.2 (3)
N2—Ti1—C11—C15	−114.7 (2)	C5—C11—C15—C14	174.4 (3)
C14—Ti1—C11—C15	37.4 (2)	Ti1—C11—C15—C14	−63.5 (2)
C13—Ti1—C11—C15	79.0 (2)	C12—C11—C15—Ti1	63.77 (19)
Cl2—Ti1—C11—C15	69.8 (2)	C5—C11—C15—Ti1	−122.1 (3)
Cl1—Ti1—C11—C15	−38.5 (2)	C13—C14—C15—C11	0.2 (4)
Cl3—Ti1—C11—C15	167.10 (18)	Ti1—C14—C15—C11	65.8 (2)
C12—Ti1—C11—C15	115.8 (3)	C13—C14—C15—Ti1	−65.6 (2)
N2—Ti1—C11—C12	129.50 (19)	N2—Ti1—C15—C11	63.58 (19)
C14—Ti1—C11—C12	−78.4 (2)	C14—Ti1—C15—C11	−116.3 (3)
C13—Ti1—C11—C12	−36.8 (2)	C13—Ti1—C15—C11	−78.6 (2)
Cl2—Ti1—C11—C12	−45.9 (2)	Cl2—Ti1—C15—C11	−137.18 (15)
Cl1—Ti1—C11—C12	−154.28 (17)	Cl1—Ti1—C15—C11	143.60 (18)
C15—Ti1—C11—C12	−115.8 (3)	Cl3—Ti1—C15—C11	−17.9 (2)
Cl3—Ti1—C11—C12	51.32 (19)	C12—Ti1—C15—C11	−37.23 (17)
N2—Ti1—C11—C5	6.3 (2)	N2—Ti1—C15—C14	179.9 (2)
C14—Ti1—C11—C5	158.4 (3)	C13—Ti1—C15—C14	37.7 (2)
C13—Ti1—C11—C5	−160.0 (3)	Cl2—Ti1—C15—C14	−20.9 (3)
Cl2—Ti1—C11—C5	−169.14 (18)	Cl1—Ti1—C15—C14	−100.1 (2)
Cl1—Ti1—C11—C5	82.5 (2)	Cl3—Ti1—C15—C14	98.4 (2)
C15—Ti1—C11—C5	121.0 (3)	C12—Ti1—C15—C14	79.1 (2)
Cl3—Ti1—C11—C5	−71.9 (2)	C11—Ti1—C15—C14	116.3 (3)
C12—Ti1—C11—C5	−123.2 (3)	C24—O1—C21—C22	17 (2)
C15—C11—C12—C13	−0.6 (3)	O1—C21—C22—C23	−14 (2)
C5—C11—C12—C13	−174.7 (3)	C21—C22—C23—C24	6(2)
Ti1—C11—C12—C13	62.5 (2)	C21—O1—C24—C23	−13 (2)
C15—C11—C12—Ti1	−63.10 (19)	C22—C23—C24—O1	4(2)
C5—C11—C12—Ti1	122.8 (3)		

### Table 1 Geometrical parameters (Å, °) of the environment of the Ti atom in the title compound compared with those of related structures

**Table:**

	(I)	(III)	(IV)	(V)	(VI)
Ti1—N2	2.153 (2)	2.163 (2)	2.357 (1)	2.274 (4)	2.261 (2)
Ti1—Cl1	2.3475 (9)	2.3513 (8)	2.3217 (4)	2.322 (2)	2.331 (1)
Ti1—Cl2	2.3377 (10)	2.3486 (8)	2.3729 (5)	2.326 (2)	2.338 (1)
Ti1—Cl3	2.3533 (9)	2.3340 (8)	2.2895 (5)	2.300 (2)	2.307 (1)
Ti1···Cp_cent_	2.030 (1)	2.036	2.025	2.035	2.047
Ti1···*PL*1	2.029 (1)	2.034 (1)	2.025	2.034	2.046
Ti1···*PL*2	0.022 (5)	0.608		0.175	0.215
N2—Ti1···Cp_cent_	111.2 (1)*^a^*	110.20	99.66	101.44	101.64
Cl1—Ti1···Cp_cent_	109.2 (1)*^a^*	110.08	116.37	116.68	113.90
Cl2—Ti1···Cp_cent_	110.69 (9)*^a^*	109.75	107.63	109.28	109.73
Cl3—Ti1···Cp_cent_	110.58 (9)*^a^*	110.93	114.71	113.76	115.56
Cl1—Ti1—N2	80.79 (7)	79.24 (6)	82.57 (2)	78.64	80.32
Cl2—Ti1—N2	138.10 (6)	140.05 (6)	152.70 (3)	149.27	148.63
Cl3—Ti1—N2	80.82 (7)	81.19 (6)	83.45 (2)	80.23	78.94
Cl2—Ti1—Cl1	85.38 (4)	86.06 (3)	85.18 (2)	87.84	87.14
Cl2—Ti1—Cl3	85.07 (4)	86.01 (3)	85.30 (2)	86.95	87.20
Cl3—Ti1—Cl1	139.98 (4)	138.52 (3)	128.55 (2)	128.07	129.12
*PL*1–*PL*2	81.0 (1)	78.335		82.491	85.895

Notes: (*a*) the angle between the Ti1—N2 bond and the normal to *PL*1; (I) this work; (III) Krut'ko *et al.* (2006); (IV) Herrmann *et al.* (1995); (V) Enders *et al.* (1997); (VI) Enders *et al.* (1996). *PL*1 and Cp_cent_ denote the C11–C15 Cp ring r.m.s. plane and centroid, respectively, while *PL*2 denotes an r.m.s. plane through the non-H atoms of a heterocyclic ring.
